# Supplementary material for: Modelling the Decamerisation Cycle of PRDX1 and the Inhibition-like Effect on Its Peroxidase Activity
Source: Antioxidants (Basel). 2023 Sep 1;12(9):1707. doi: 10.3390/antiox12091707 (PMC10525498; doi:10.3390/antiox12091707)
Supplement: Supplementary file 1 [file antioxidants-12-01707-s001.zip › antioxidants-2497867-supplementary.pdf]

Supplementary Material:
Modelling the decamerisation cycle of PRDX1 and the inhibition-like effect on its peroxidase activity

Christopher J. Barry[1], Ché S. Pillay[2], and Johann M. Rohwer[1*]

[1]Laboratory for Molecular Systems Biology, Dept. of Biochemistry, Stellenbosch University, Stellenbosch, South Africa
[2]School of Life Sciences, Univ. of KwaZulu-Natal, Pietermaritzburg, South Africa
[*]For correspondence: jr@sun.ac.za

Contents

Data and code availability 2

S1 Models 2

S2 Processing isothermal titration calorimetry data 4

S3 Algorithm for enumerating molecular states of peroxiredoxin 6

S4 Reproducing Benfeitas et al. 2014 analyses 9

S5 Time to equilibrium after dilution of PRDX1 14

S6 Oxidation state analysis 15

S7 Testing an alternative dissociation scheme 17

Data and code availability

All data and code required to reproduce the simulations and results presented in this paper are available from GitHub: https://github.com/Rohwer-Lab/Barry2023

The GitHub page also provides instructions for running the simulations online and for setting up a local computational environment on your own device.

S1 Models

The various models used in this study are listed in **Table S1** and their associated reactions and parameters are summarised in **Table S2**.

Table S1: Descriptions of the models used in this study.

**Model**	**Description**
Prx decamerisation	Prx association and dissociation reactions.
ITC Prx with decamerisation	Prx association and dissociation reactions and a trigger for periodically "injecting" Prx.
RBC Prx Model A [1]	Red blood cell model of H_2O_2 metabolism by several enzymes as described in Benfeitas et al 2014.
RBC Prx Model B [1]	Red blood cell model of H_2O_2 metabolism by several enzymes with Prx inhibition by sequestration as described in Benfeitas et al. 2014.
RBC Prx Model A with decamerisation	Red blood cell model of H_2O_2 metabolism by several enzymes as described in Benfeitas et al. 2014, augmented with Prx decamer association, dissociation, and peroxidase reactions.
Prx decamerisation dilution	Prx association and dissociation reactions and a trigger for diluting Prx.
HRP Prx	Horse radish peroxidase competition assay model containing peroxidase reactions for HRP as well as Prx.
HRP Prx with decamerisation	Horse radish peroxidase competition assay model containing peroxidase reactions for HRP and Prx, as well as Prx decamer association, dissociation, and peroxidase reactions.
Prx cycle	Prx, Trx, H_2O_2, and NADPH species with reactions for Prx dimer peroxidase, Trx regeneration by TRR, and sulfinic Prx regeneration by Srx.
Prx cycle with decamerisation	Prx, Trx, H_2O_2, and NADPH species with reactions for Prx dimer peroxidase, Prx decamer peroxidase, Trx regeneration by TRR, and sulfinic Prx regeneration by Srx, as well as Prx decamer association and dissociation reactions.

Table S2: Stoichiometry, rate equations and model parameters used in the simulations. Peroxiredoxin (Prx) sites are represented as: SH, reduced; SOH, sulfenilated; SS, disulfide bridge; SO2H, sulfinilated. Models: α, Prx decamerisation; β, ITC with Prx decamerisation; γ, Prx decamerisation dilution; ε, HRP-Prx; ζ, HRP-Prx with decamerisation; η, Prx cycle; θ, Prx cycle with decamerisation.

**Model**							**Stoichiometry**	**Rate or trigger condition**	**Value**
α	β	γ	ε	ζ	η	θ			
							Decamer association and dissociation		
α	β	γ	ε	ζ		θ	$Prx_{dec.\ all\ SH} \rightarrow 5 \cdot SH\_SH$ †	$Prx_{dec.\ all\ SH} \cdot k_{off} - SH\_SH^5 \cdot k_{on}$	k_{off} = 0.055 s^{-1} [this study]
									k_{on} = 0.050 $\mu M^{-4} \cdot s^{-1}$ [this study]
				ζ		θ	$Prx_{dec.\ with\ SS} \rightarrow 5 \cdot Prx_{dim.}$ † *	$k_{disso} \cdot Prx_{dec.\ with\ SS}$	k_{disso} = 10000 s^{-1}
							Sulfenilation		
				ζ		θ	$Prx_{dec.\ with\ SH} \rightarrow Prx_{dec.\ with\ SOH}$ †	$nSH_{sites} \cdot k_{sulfe} \cdot Prx_{dec.\ with\ SH} \cdot H_2O_2$	$k_{sulfe\ dec}$ = 100 $\mu M^{-1} \cdot s^{-1}$ [2]
			ε	ζ	η	θ	$SH\_SH + H_2O_2 \rightarrow SH\_SOH$	$2 \cdot k_{sulfe} \cdot SH\_SH \cdot H_2O_2$	$k_{sulfe\ dim}$ = 1.0 $\mu M^{-1} \cdot s^{-1}$
			ε	ζ	η	θ	$SH\_SOH + H_2O_2 \rightarrow SOH\_SOH$	$k_{sulfe} \cdot SH\_SOH \cdot H_2O_2$	
			ε	ζ	η	θ	$SH\_SS + H_2O_2 \rightarrow SOH\_SS$	$k_{sulfe} \cdot SH\_SS \cdot H_2O_2$	
			ε	ζ	η	θ	$SH\_SO2H + H_2O_2 \rightarrow SOH\_SO2H$	$k_{sulfe} \cdot SH\_SO2H \cdot H_2O_2$	
							Sulfinilation		
			ε	ζ	η	θ	$SH\_SOH + H_2O_2 \rightarrow SH\_SO2H$	$k_{sulfi} \cdot SH\_SOH \cdot H_2O_2$	k_{sulfi} = 0.012 $\mu M^{-1} \cdot s^{-1}$ [3]
			ε	ζ	η	θ	$SOH\_SOH + H_2O_2 \rightarrow SOH\_SO2H$	$2 \cdot k_{sulfi} \cdot SOH\_SOH \cdot H_2O_2$	
			ε	ζ	η	θ	$SOH\_SS + H_2O_2 \rightarrow SS\_SO2H$	$k_{sulfi} \cdot SOH\_SS \cdot H_2O_2$	
			ε	ζ	η	θ	$SOH\_SO2H + H_2O_2 \rightarrow SO2H\_SO2H$	$k_{sulfi} \cdot SOH\_SO2H \cdot H_2O_2$	
							Disulphide-bridge formation		
				ζ		θ	$Prx_{dec.\ with\ SOH} \rightarrow Prx_{dec.\ with\ SS}$ †	$nSOH_{sites} \cdot k_{disulf\ form} \cdot Prx_{dec.\ with\ SOH}$	$k_{disulf form}$ = 1.7 s^{-1} [3]
			ε	ζ	η	θ	$SH\_SOH \rightarrow SH\_SS$	$k_{disulf\ form} \cdot SH\_SOH$	
			ε	ζ	η	θ	$SOH\_SOH \rightarrow SOH\_SS$	$2 \cdot k_{disulf\ form} \cdot SOH\_SOH$	
			ε	ζ	η	θ	$SOH\_SS \rightarrow SS\_SS$	$k_{disulf\ form} \cdot SOH\_SS$	
			ε	ζ	η	θ	$SOH\_SO2H \rightarrow SS\_SO2H$	$k_{disulf\ form} \cdot SOH\_SO2H$	
							Disulphide-bridge reduction		
					η	θ	$SH\_SS + TrxSH \rightarrow SH\_SH + TrxSOH$	$k_{disulf\ red} \cdot SH\_SS \cdot TrxSH$	$k_{disulf\ red}$ = 0.21 $\mu M^{-1} \cdot s^{-1}$ [2]
					η	θ	$SOH\_SS + TrxSH \rightarrow SH\_SOH + TrxSOH$	$k_{disulf\ red} \cdot SOH\_SS \cdot TrxSH$	
					η	θ	$SS\_SS + TrxSH \rightarrow SH\_SS + TrxSOH$	$2 \cdot k_{disulf\ red} \cdot SS\_SS \cdot TrxSH$	
					η	θ	$SS\_SO2H + TrxSH \rightarrow SH\_SO2H + TrxSOH$	$k_{disulf\ red} \cdot SS\_SO2H \cdot TrxSH$	
							Thioredoxin regeneration		
					η	θ	$TrxSOH + NADPH \rightarrow NADP + TrxSH$	$k_{cat} \cdot TrxR \cdot (NADPH \cdot TrxSOH)/(TrxSOH \cdot NADPH + K_{M,NADPH,TrxR} \cdot TrxSOH + NADPH \cdot K_{M,TrxSOH} + K_{M,NADPH,TrxR} \cdot K_{M,TrxSOH})$	$TrxR = \frac{V_{Max,TrxR}}{k_{cat}}$ = 0.14 μM [1] SM
									k_{cat} = 73 s^{-1} [1] SM
									$K_{M,NADPH,TrxR}$ = 6.0 μM [4]
									$K_{M,TrxSOH,TrxR}$ = 1.83 μM [5]
							HRP oxidation		
			ε	ζ			$HRP + H_2O_2 \rightarrow compound\_I$	$k_{HRP} \cdot HRP \cdot H_2O_2$	k_{HRP} = 11.0 $\mu M^{-1} \cdot s^{-1}$ [2]
							ITC injection		
	β						$Prx_{dec.} \rightarrow Prx_{dec.} + inj_{dec.}$	$\frac{2 \cdot \pi \cdot cos(time_{sim} - 1)}{inj_{interval}} > 0.999999999$	$inj_{interval}$ = 200 s (based on [6])
	β						$Prx_{dim.} \rightarrow Prx_{dim.} + inj_{dim.}$	$\frac{2 \cdot \pi \cdot cos(time_{sim} - 1)}{inj_{interval}} > 0.999999999$	
	β						$vol_{cell} \rightarrow vol_{cell} + vol_{inj}$	$\frac{2 \cdot \pi \cdot cos(time_{sim} - 1)}{inj_{interval}} > 0.999999999$	$vol_{inj.}$ = 1.6 μl [6]
							Conserved moieties		
α	β	γ	ε	ζ	η	θ	$Prx_{dim.} = SH\_SH + SH\_SOH + SH\_SS + SH\_SO2H + SOH\_SOH + SOH\_SS + SOH\_SO2H + SS\_SS + SS\_SO2H + SO2H\_SO2H$		
				ζ		θ	$Prx_{total\ (dim.)} = 5 \cdot Prx_{dec.} + Prx_{dim.}$ †		
			ε	ζ			$HRP_{total} = HRP + compound\_I$		
					η	θ	$Trx_{total} = TrxSH + TrxSOH$		

† $Prx_{dec.}$ is a placeholder for the multitude of Prx decamer species.
* specific $Prx_{dim.}$ product species depends on the dissociating $Prx_{dec.}$.

S2 Processing isothermal titration calorimetry data

The ITC apparatus has several limitations, which need to be considered when analysing the heat traces. The problem of extracting an exponential decay signal from the pulse signal detected by a delayed receiver has been solved by a Laplace transform using Tian's equation [7, 8]:

$$b_{deconv}(t) = g_{experimental}(t) + \frac{dg_{experimental}(t)}{dt} \cdot \tau_r$$

with b_{deconv} as the real µcal/sec, $g_{experimental}$ as the reading of µcal/sec from the adiabatic cell and τ_r as the time delay between the heat release by reagents and the adiabatic cell reading. The ITC trace of Figure 1C in [6] was transformed by finding the derivative of each data point and using a τ_r of 20 s from the documentation of the VP (Microcal, Northampton, MA) calorimeter. **Figure S1 a** shows the transformation of a single peak with a fast initial reaction where the majority of the heat of dissociation is released. This reaction is followed by a slower reaction which takes place over the rest of the injection period. The curve between these two reactions suggests a third reaction may be present. In order to compensate for errors in determining ΔH, the data were normalised to the area under the curve of each injection peak. Starting with the full trace (**Figure S1 b**), data after the CTC injection were removed, yielding the final peaks used for fitting (**Figure S1 c**). The digitised data were interpolated for each data point to facilitate fitting to *in silico* data.

When using Tian's equation, the data points taken between the injection and τ_r are unreliable [9]. Furthermore, the process of interpolating data can introduce a heat peak a few seconds before each injection. To avoid these issues, the data points between 5 s before each injection and 20 s after each injection were filtered out, leaving a final trace that was used for fitting (**Figure S1 d**).

Several complications occur in ITC experiments, which need to be taken into consideration when fitting *in silico* to *in vitro* data. ITC injections produce heat peaks independently of chemical reactions, called heat of dilution, which are usually quantified in a control experiment that includes only one of the reaction components, and subsequently subtracted from the data. This control experiment is not viable when studying protein dissociation as a single component of a dissociation cannot be isolated. The approach followed by Barranco-Medina and co-workers in their experiments was to assume that no dissociation occurs after the CTC and to subtract those peaks from the data before analysis [6]. This was not required in our simulations, as we did not explicitly model heat of dilution. However, small amounts of heat generation can be seen in **Figure 3 e** and **f** of the main text. These are due to residual dissociation still occurring above the CTC; when modelling such an equilibrium with mass action, the concentration of dimers will keep increasing as more concentrated protein solution is injected, implying that dissociation still occurs, albeit at a much reduced rate.

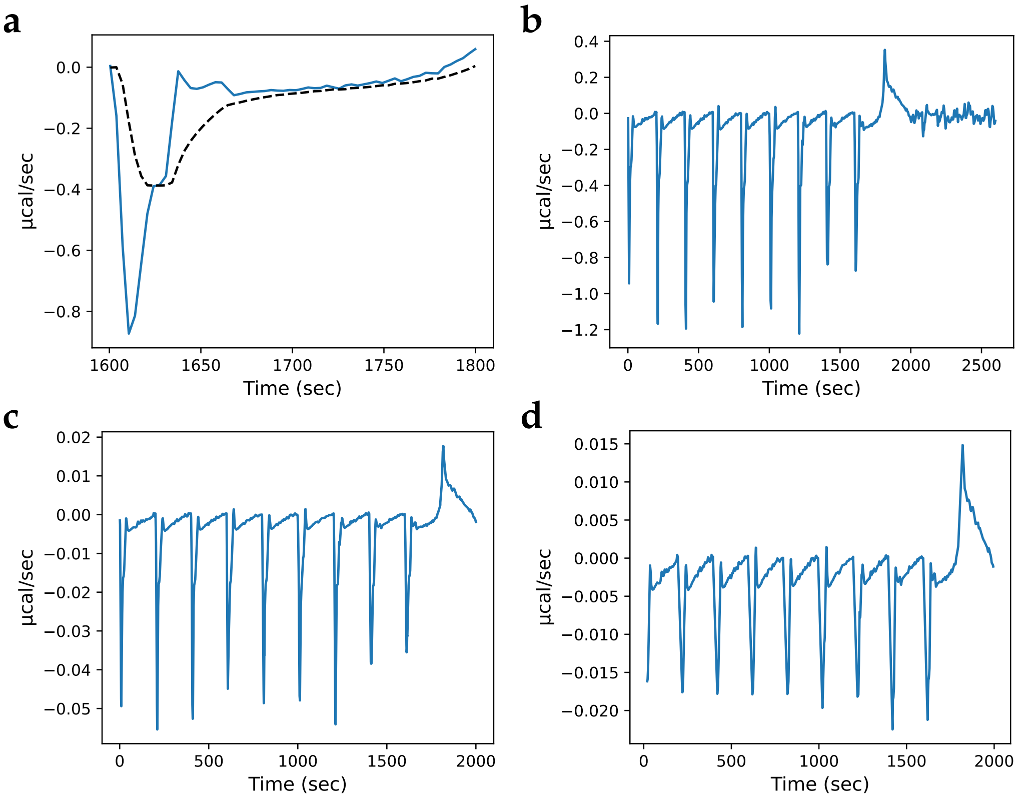

Figure S1: Processing of isothermal titration calorimetry data digitised from Figure 1C in Barranco Medina et al. [6]. **(a)** Comparison of (- -) the original heat trace and (—) the Laplace transform of one heat peak as per Tian's equation [7, 8]. **(b)** Full trace of Laplace-transformed heat curves. **(c)** Data from (b) normalized to the area under the curve. **(d)** Data from (c) with data acquired between injection and machine delay filtered out (see text).

S3 Algorithm for enumerating molecular states of peroxiredoxin

A Python script was used to enumerate the species and peroxidase reactions of a Prx decamer by recursively "reacting" decamers and collating the degeneracy. The justification for the each type of degeneracy can be found in the main text. The goal of this script was to create a plain text file that contained the minimum set of rate and stoichiometric equations to describe the peroxidase reactions and subsequent dissociation of the Prx decamer. Below the logic of the script is described as a series of steps with an example.

Step 1: React substrate

The script begins with a substrate decamer and a reaction as `<Input>` and produces several product decamers as `<Outputs>` by "reacting" each viable site in the `<Input>` substrate decamer by the `<Input>` reaction. The reactions considered were: sulfenilation, $SH \rightarrow SOH$; condensation, $SOH \rightarrow SS$; or sulfinilation, $SOH \rightarrow SO2H$. As an example, we show how the sites of a particular substrate decamer (*SH_SH__SH_SOH__SH_SH__SOH_SOH__SOH_SOH*) are "reacted" by sulfenilation (**Figure S2 a**).

Step 2: Mirror symmetry

Each `<Output>` of step 1 becomes an `<Input>` for step 2. Each dimer of the `<Input>` decamer is reoriented such that left-hand site conforms to the priority rules: $SH > SOH > SS > SO2H$. By way of example, this is illustrated for the last `<Output>` from step 1, with the reoriented sites highlighted in blue (**Figure S2 b**). We assume that there is no cooperativity between dimer sites and their adjacent dimers. Each `<Input>` of step 2 results in one `<Output>` (perhaps unchanged), which becomes an `<Input>` for step 3.

Step 3: Rotational and Planar symmetry

During step 3, several candidate decamers are generated from the `<Input>` decamer but only one is advanced as the `<Output>`. To generate these candidates, the `<Input>` decamer is "rotated" through each possible orientation (**Figure S2 c**, left-hand branch). Each of the generated candidate decamers is then scored by summing the scores of its constituent dimers according to

$$score = \sum_{n=1}^{5} n \times 10^{dim_{rank}},$$

where n is the position of the dimer in the decamer (left-to-right) and dim_{rank} a 1–9 priority rank of each dimer according to **Table S3**.

Next, candidates are generated based on planar symmetry (**Figure S2 c**, right-hand branch). The `<Input>` decamer is "flipped" and reoriented for mirror symmetry as in step 2, and after this, the decamer follows the same rotation and scoring procedure as above.

After completion of step 3, each `<Input>` produces up to 10 candidate decamers of which the highest scoring candidate is selected as `<Output>`. This scoring system ensures that all orientations of a decamer that can be considered identical by the various forms of symmetry will have the same `<Output>` species.

Step 4: Collate degeneracy

As `<Inputs>`, step 4 takes the complete list of `<Outputs>` from step 1 after they have been individually processed through steps 2 and 3. Next, the degeneracy of the resulting

Table S3: The rank of dimers used for scoring candidate decamers. Note the lack of score for *SS_SS* as decamers were modelled as dissociating immediately after formation of a single disulphide-bridge.

**dimer**	**Rank**
SH_SH	1
SH_SOH	2
SH_SS	3
SH_SO2H	4
SOH_SOH	5
SOH_SS	6
SOH_SO2H	7
SS_SO2H	8
SO2H_SO2H	9

`<Outputs>` is collated so that final set contains the unique reactions from the original `<Inputs>` as well a record of the number of multiples of each unique reaction (**Figure S2 d**). Later, the number of multiples will be used as a statistical factor in the rate equations for the associated reaction.

Step 5: Recursion

Finally, the process is applied recursively for sulfenilation and sulfinilation reactions but not for condensation reactions. To achieve this, the species in the `<Output>` of step 4 are recursively fed back into into the script until there are no more viable sites for any reaction `<Input>` (sulfenilation or sulfinilation) in step 1. The script of the condensation reaction is not run recursively since the decamers were modelled as dissociating immediately after formation of a single disulphide bridge. Instead, the dissociation reactions for these species are enumerated.

Rate and stoichiometric reactions

With the substrate and product pairs enumerated, we were able to generate stoichiometric and rate equations for each reaction of the Prx decamer. These equations were incorporated in `*.psc` model files and formalised into ordinary differential equations for simulation using the PySCeS software [10].

Running this script for sulfenilation, sulfinilation and condensation reactions resulted in a network of some 9990 reactions, which was computationally prohibitive. We repeated the process for the sulfenilation and condensation reactions only, which resulted in a set of 270 reactions. As elaborated on in the main text, no kinetics are available for the difference between the sulfinilation rate constants of dimer and decamer, and moreover, disulphide-bridge formation is likely the main driver of decamer dissociation. We thus assumed that the reduced reaction set encompassed the relevant reactions of the Prx decamer, while still allowing for computationally manageable simulations of our analyses. Sulfinilation reactions, the sulfinilated forms of Prx, and their reduction by Srx were thus modelled only at the dimer level.

a

<Input> SH_SH__ SH_SOH__ SH_SH__ SOH_SOH__ SOH_SOH

sulfenilation

<Outputs>

SH_SH__ SH_SOH__ SH_SOH__ SOH_SOH__ SOH_SOH
SH_SH__ SH_SOH__ SOH_SH__ SOH_SOH__ SOH_SOH
SH_SH__ SOH_SOH__ SH_SH__ SOH_SOH__ SOH_SOH
SH_SOH__ SH_SOH__ SH_SH__ SOH_SOH__ SOH_SOH
SOH_SH__ SH_SOH__ SH_SH__ SOH_SOH__ SOH_SOH

b

<Input> SOH_SH__ SH_SOH__ SH_SH__ SOH_SOH__ SOH_SOH

<Output> SH_SOH__ SH_SOH__ SH_SH__ SOH_SOH__ SOH_SOH

c

<Input> SH_SOH__ SH_SOH__ SH_SH__ SOH_SOH__ SOH_SOH

Left-hand side:

SH_SOH__ SH_SOH__ SH_SH__ SOH_SOH__ SOH_SOH

SH_SOH__ SH_SOH__ SH_SH__ SOH_SOH__ SOH_SOH: 900330
SOH_SOH__ **SH_SOH**__ SH_SOH__ SH_SH__ SOH_SOH: 600540
SOH_SOH__ SOH_SOH__ **SH_SOH**__ SH_SOH__ SH_SH: 300750
SH_SH__ SOH_SOH__ SOH_SOH__ **SH_SOH**__ SH_SOH: 500910
SH_SOH__ SH_SH__ SOH_SOH__ SOH_SOH__ **SH_SOH**: 700620

Right-hand side:

SOH_SOH__ SOH_SOH__ SH_SH__ SOH_SH__ **SOH_SH**

SOH_SOH__ SOH_SOH__ SH_SH__ SH_SOH__ SH_SOH

SOH_SOH__ SOH_SOH__ SH_SH__ SH_SOH__ SH_SOH: 300930
SH_SOH__ **SOH_SOH**__ SOH_SOH__ SH_SH__ SH_SOH: 500640
SH_SOH__ SH_SOH__ **SOH_SOH**__ SOH_SOH__ SH_SH: 700350
SH_SH__ SH_SOH__ SH_SOH__ **SOH_SOH**__ SOH_SOH: 900510
SOH_SOH__ SH_SH__ SH_SOH__ SH_SOH__ **SOH_SOH**: 500820

<Output> SH_SH__ SH_SOH__ SH_SOH__ SOH_SOH__ SOH_SOH

d

<Inputs>

SH_SH__ SH_SOH__ SH_SOH__ SOH_SOH__ SOH_SOH
SH_SH__ SH_SOH__ SH_SOH__ SOH_SOH__ SOH_SOH
SH_SH__ SOH_SOH__ SH_SH__ SOH_SOH__ SOH_SOH
SH_SH__ SH_SOH__ SH_SOH__ SOH_SOH__ SOH_SOH
SH_SH__ SH_SOH__ SH_SOH__ SOH_SOH__ SOH_SOH

<Outputs>

4 × SH_SH__ SOH_SOH__ SH_SH__ SOH_SOH__ SOH_SOH
1 × SH_SH__ SH_SOH__ SH_SOH__ SOH_SOH__ SOH_SOH

Figure S2: Example of the algorithm for enumerating the molecular states of decameric Prx. **(a)** Step 1, the SH sites in the substrate decamer are "reacted" to SOH (shown in blue). **(b)** Step 2, the "SOH_SH" dimer in the decamer is reoriented so that the left site follows the priority order of: *SH > SOH > SS > SO2H*. **(c)** Step 3 left-hand side, the <Input> decamer is "rotated" through each possible orientation to generate 5 candidate decamers, each of which is scored by summing the scores of its constituent dimers. Right-hand side, the <Input> decamer is "flipped", corrected for mirror symmetry as in (b), and then candidates are enumerated and scored as per the left-hand side. **(d)** Step 4, after processing by (b) and (c), the <Outputs> of (a) are collated for degeneracy.

S4 Reproducing Benfeitas et al. 2014 analyses

Benfeitas and co-workers [1] published several model variants of the human PRDX2 system in the red blood cell together with analyses of the system. We used these models to compare the Prx system with and without Prx decamer association and dissociation in an *in vivo* context.

The code for a version of the RBC model of the PRDX2 system with an inhibitor (most closely corresponding to Model B in the original publication) was supplied in the supplementary material of [1]. We converted this model to the `*.psc` file format for use with the PySCeS software and renamed the species and reactions to maintain consistency with other models in this work. In order to replicate the analyses of "Model B" in [1], it was necessary to add a hydrogen peroxide bolus reaction. To replicate "Model A" in [1], the inhibition reactions in "Model B" were disabled. Finally, we created "Model A with decamerisation" by augmenting "Model A" with the decamerisation reactions determined by the algorithm described in **Figure S2** as well as the parameters summarised in **Table S2**.

We reproduce here several of the analyses in Benfeitas et al. [1], which compared "Model A" and "Model B" and included our "Model A with decamerisation". The first point to note is that Figures 4–7 in [1] are nearly perfectly replicated in **Figure S3**–**Figure S6**, respectively, which validates our implementation of the original model code. The only exception, and one we are unable to explain, was GSH potential following a hydrogen peroxide pulse (**Figure S5 b**) where our simulations followed the same trend as the original publication but had different numerical values. Second, the results of our Model A with decamerisation most often agreed with the original Model A. A notable exception is the oxidation state of the Prx dimers (**Figure 6** in the main paper, as well as **Figure S3 b** and **d**), where Model A with decamerisation agrees more closely with Model B. The dimer oxidation state is the most important experimental observation against which the original model was validated, and this is the basis for our conclusion that Prx decamerisation can explain the inhibitory effect, which was included in Model B with an inhibited form for which there is no direct experimental evidence (see also **Section 3.7** and **Discussion** in the main paper).

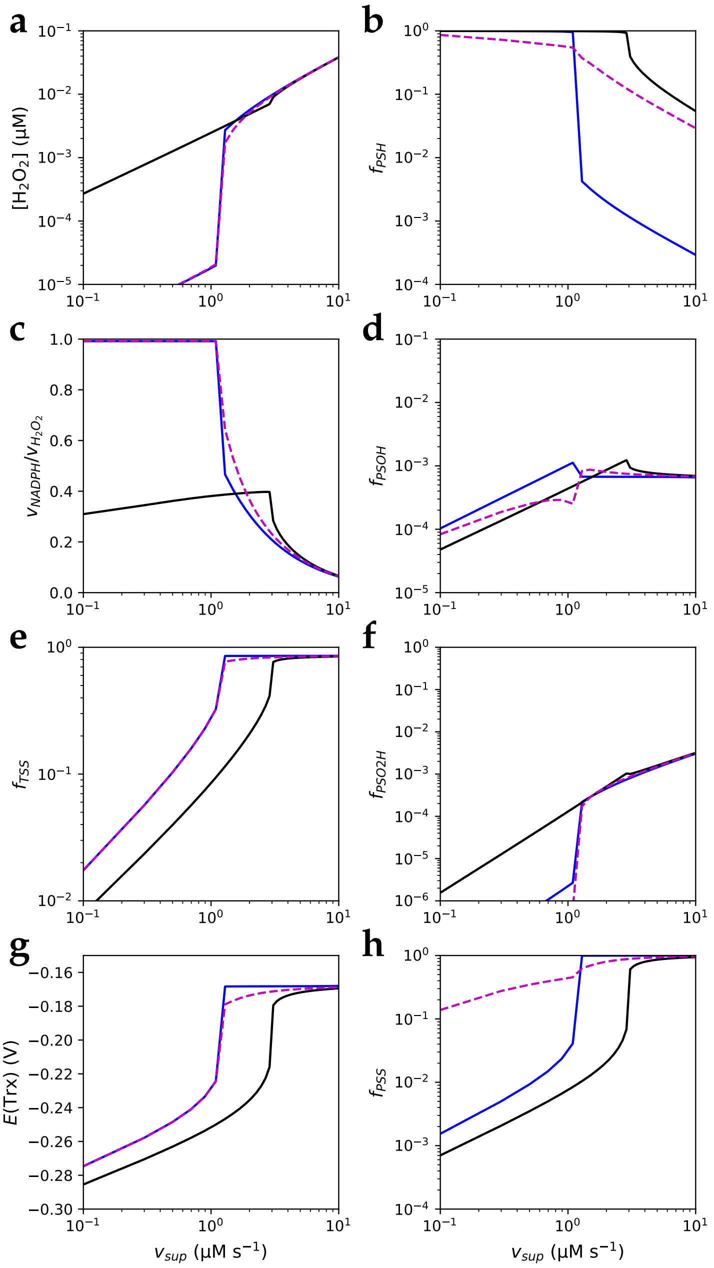

Figure S3: Reproduction of Figure 4 in [1]: Steady-state responses of alternative model designs of the Prx2 redox cycle in hydrogen peroxide metabolism to changes in H_2O_2 supply. **(a)** Intracellular hydrogen peroxide concentration, **(b)** fraction of reduced Prx, **(c)** ratio of NADPH oxidation rate to hydrogen peroxide consumption rate, **(d)** fraction of sulfenilated Prx, **(e)** fraction of thioredoxin with disulphide bridge, **(f)** fraction of sulfinilated Prx, **(g)** thioredoxin redox potential, and **(h)** fraction of Prx with disulphide bridge. (—) Model A; (—) Model B; (- -) Model A with decamerisation.

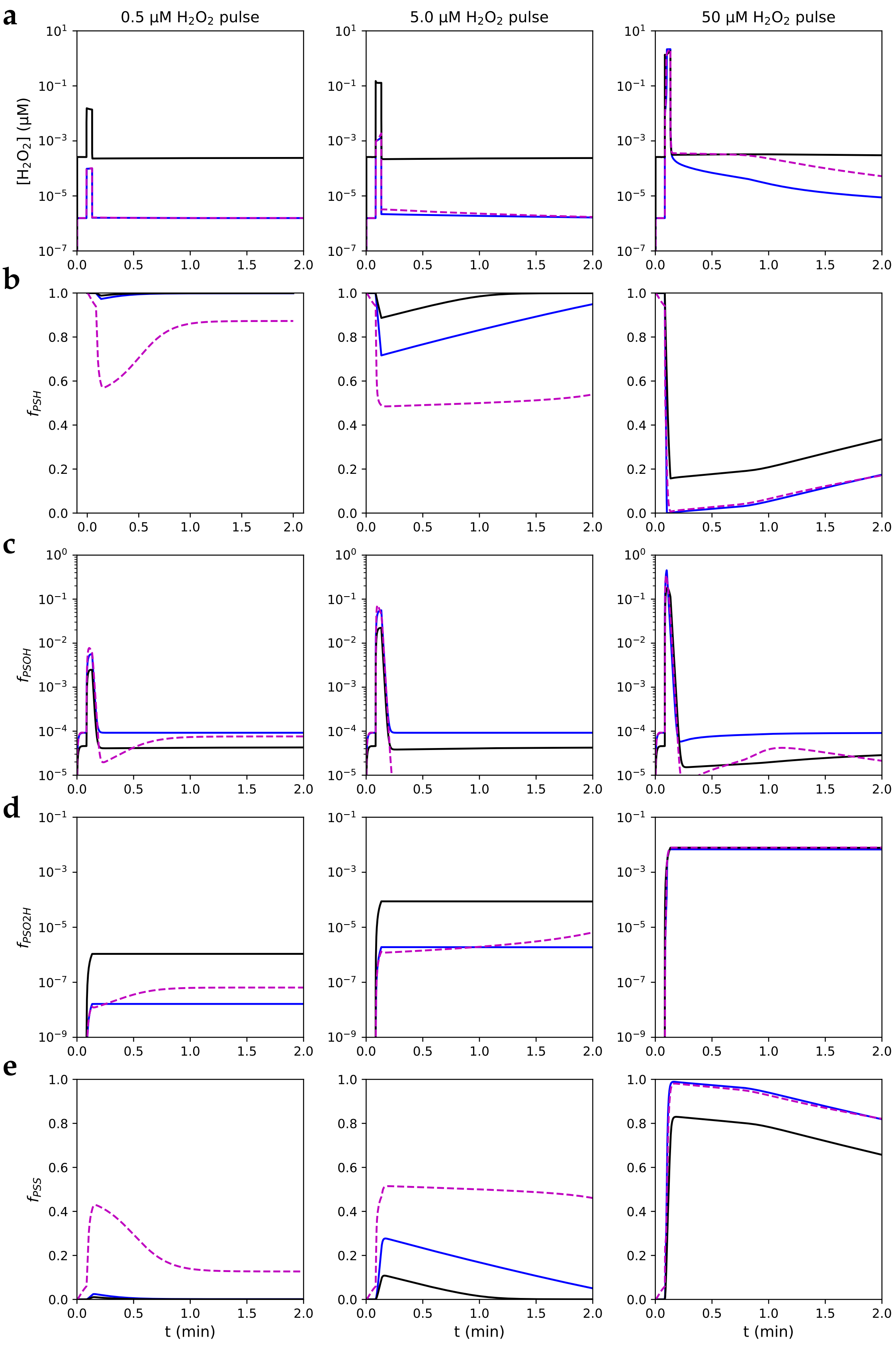

Figure S4: Reproduction of Figure 5 in [1]: Responses of intracellular hydrogen peroxide concentration and PRDX2 oxidation states to 3 s extracellular H_2O_2 pulses. **(a)** Intracellular hydrogen peroxide concentration, **(b)** fraction of reduced Prx, **(c)** fraction of sulfenilated Prx, **(d)** fraction of sulfinilated Prx, and **(e)** fraction of Prx with disulphide bridge. (—) Model A; (—) Model B; (- -) Model A with decamerisation.

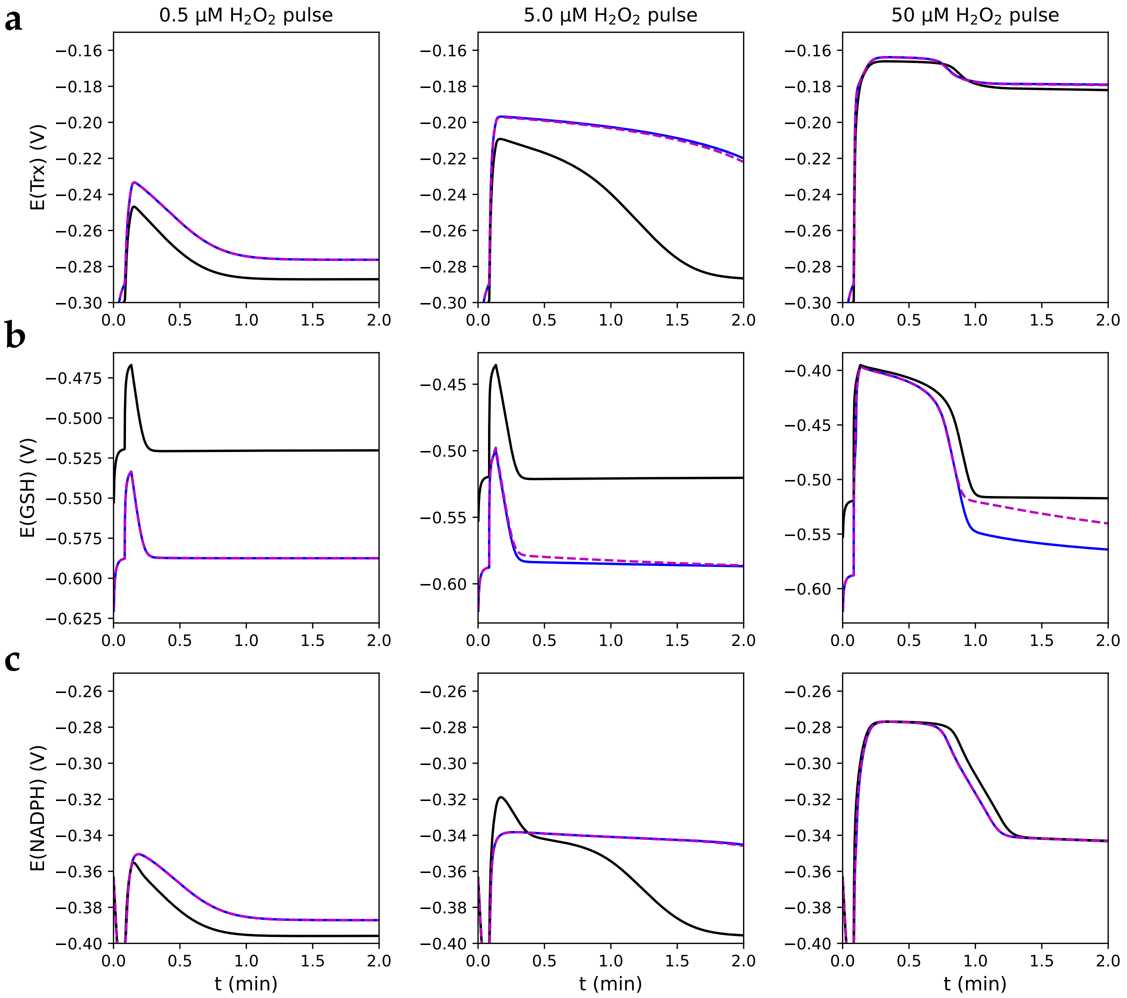

Figure S5: Reproduction of Figure 6 in in [1]: Responses of the potentials of redox pools to 3 s extracellular H_2O_2 pulses. **(a)** Thioredoxin redox potential, **(b)** glutathione redox potential, and **(c)** NADPH redox potential. (—) Model A; (—) Model B; (- -) Model A with decamerisation.

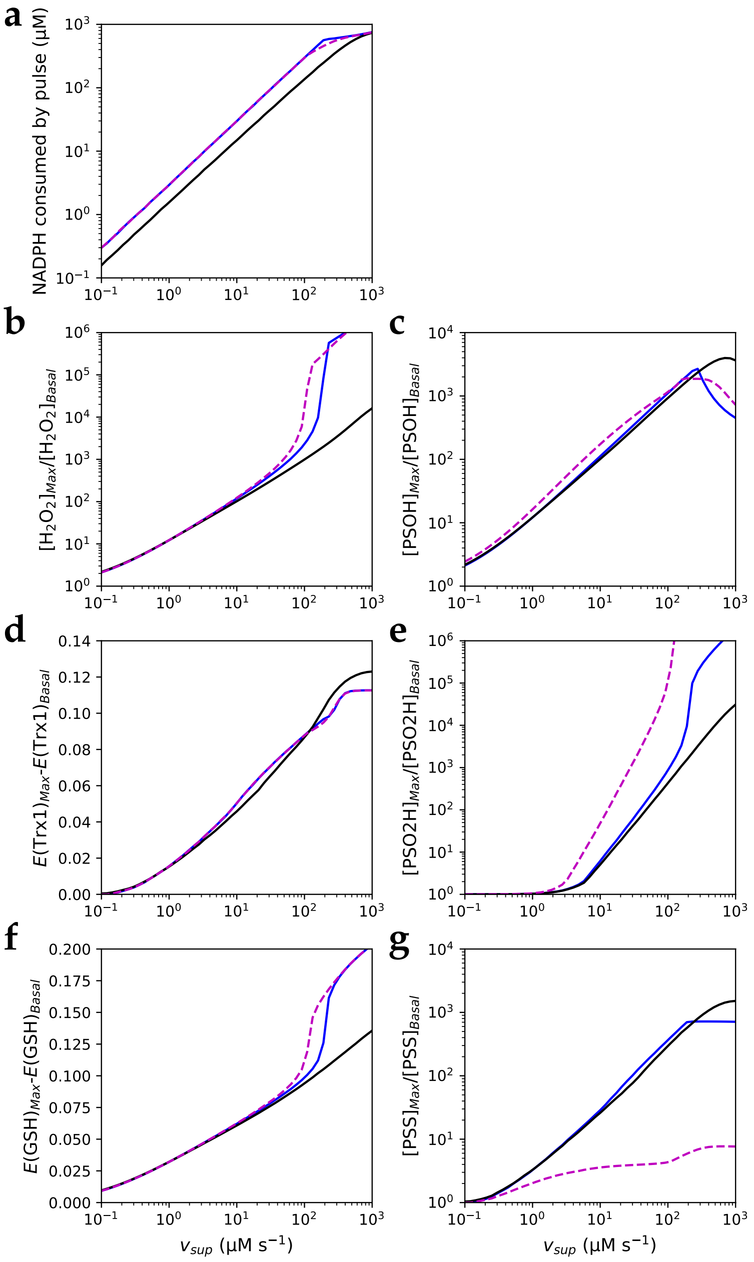

Figure S6: Reproduction of Figure 7 in [1]: Responses of intracellular variables as a function of the amplitude of a 3 s H_2O_2 supply pulse. **(a)** Total NADPH consumed, **(b)** ratio of maximal to basal intracellular hydrogen peroxide concentration, **(c)** ratio of maximal to basal sulfenilated Prx, **(d)** difference between maximal and basal thioredoxin 1 redox potential, **(e)** ratio of maximal to basal sulfinilated Prx, **(f)** difference between maximal and basal glutathione redox potential, and **(g)** ratio of maximal to basal Prx with disulphide bridge. (—) Model A; (—) Model B; (- -) Model A with decamerisation.

S5 Time to equilibrium after dilution of PRDX1

Diluting a solution of Prx lowers the concentration and thereby causes Prx decamers to dissociate into dimers. Using simulations with our determined rate constants for the Prx decamer-dimer transition, we show that the time to reach equilibrium increases for greater dilution factors and is inversely related to the original solution concentration (**Figure S7**).

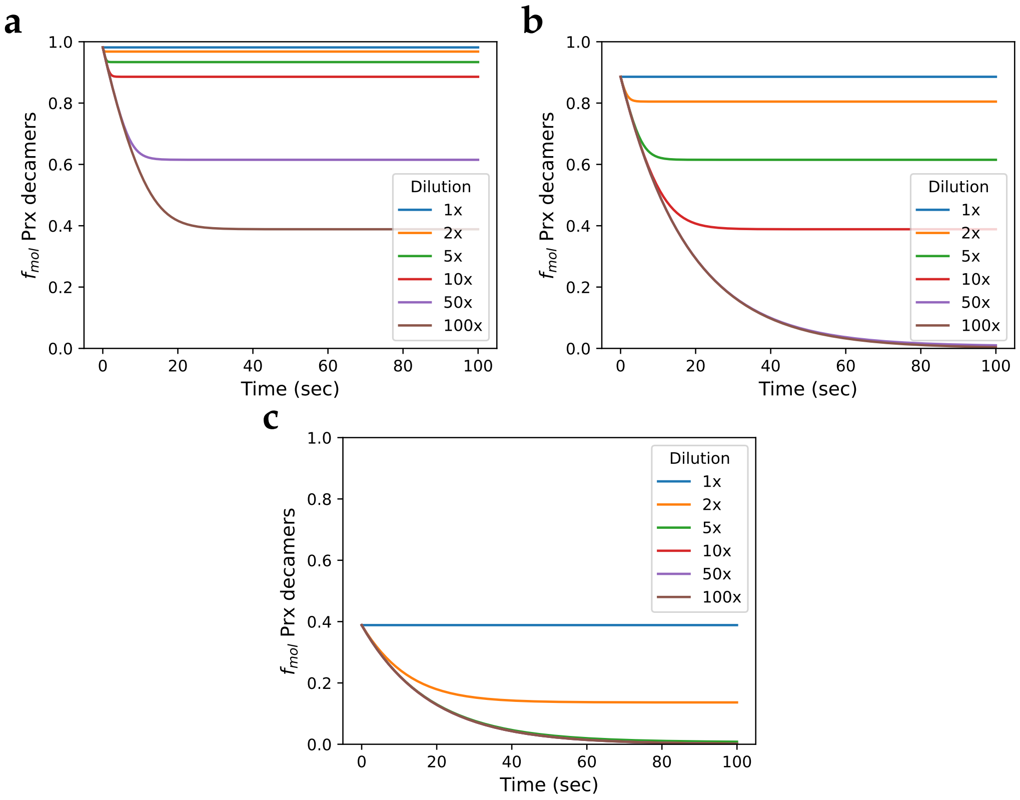

Figure S7: The time for Prx to reach equilibrium following dilution of **(a)** 100 μM, **(b)** 10 μM, **(c)** 1 μM solutions. The dilution factor (fold-dilution) is indicated by the colours in the key.

S6 Oxidation state analysis

To investigate possible causes of the reduction of peroxidase activity introduced when the dimer-decamer transition is incorporated, we calculated the disequilibrium ratio Q/K_d, with the reaction quotient Q defined as: as:

$$Q = \frac{SH\_SH^5}{Prx_{dec.reduced}} \tag{1}$$

The fraction of reduced sites in fully reduced dimers was calculated as:

$$f_{sites}(\text{SH in SH\_SH dimer}) = \frac{2 \times SH\_SH}{Total\ Prx_{sites}} \tag{2}$$

and the reaction of reduced sites in hetero-oxidised as:

$$f_{sites}(\text{SH in hetero-oxidised dimer}) = \frac{SH\_SOH + SH\_SS}{Total\ Prx_{sites}} \tag{3}$$

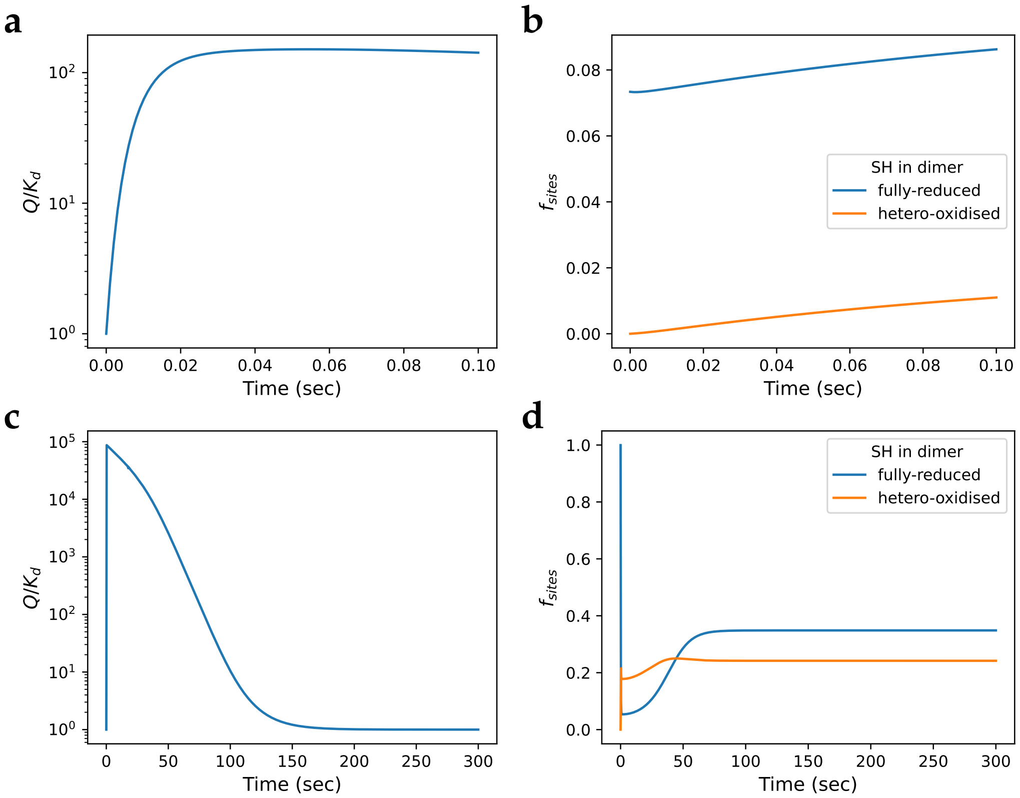

Figure S8: Reduced Prx sites are locked in hetero-oxidised dimers and the pool of fully-reduced dimers is larger than expected from the reduced dimer-decamer equilibrium. **(a)** Disequilibrium ratio and **(b)** the fraction of SH sites in fully-reduced and hetero-oxidised dimers during the course of an HRP assay in **Figure 7 a** (1.7 μM trace) of the main text. **(c)** Disequilibrium ratio and **(d)** the fraction of SH sites in fully-reduced and hetero-oxidised dimers during the course of an NADPH assay in **Figure 7 c** (0.1 μM trace) of the main text.

The disequilibrium ratio for the decamer-dimer transition, as well as the fraction of reduced sites in fully reduced *vs.* hetero-oxidised dimers, were calculated for selected simulations of the HRP assay and NADPH assay. While the system was under hydrogen peroxide stress, the proportion of the dimeric reduced Prx pool was consistently greater than that of the same system at rest, i.e. $Q/K_{eq} > 1$ (**Figure S8** **a** and **c**). Furthermore, hetero-oxidised dimers are unable to form decamers and therefore the reduced sites contained in them will have the corresponding low peroxidase activity of dimeric Prx. The fraction of hetero-oxidised dimers increased under hydrogen peroxide load (**Figure S8** **b** and **d**).

S7 Testing an alternative dissociation scheme

We replicated our RBC, HRP assay and NADPH assay simulations using an extended model that allowed for the formation of up to five disulphide bridges on a decamer (one per dimer), along with sulfenilation reactions for decamers featuring disulphide bridges. Notably, we observed that this revised scheme yielded outcomes comparable to our original model, which solely considered the formation of a single disulphide bridge without sulfenilation after disulphide bridge formation (**Figure S9**). One exception was the HRP assays, where the inclusion of sulfenilation after disulphide-bridge formation led to an increase in peroxidase activity for the mixed-activity model, increasing the rate constant from 91 $\mu M^{-1} \cdot s^{-1}$ to 148 $\mu M^{-1} \cdot s^{-1}$. This is discussed further in the main text.

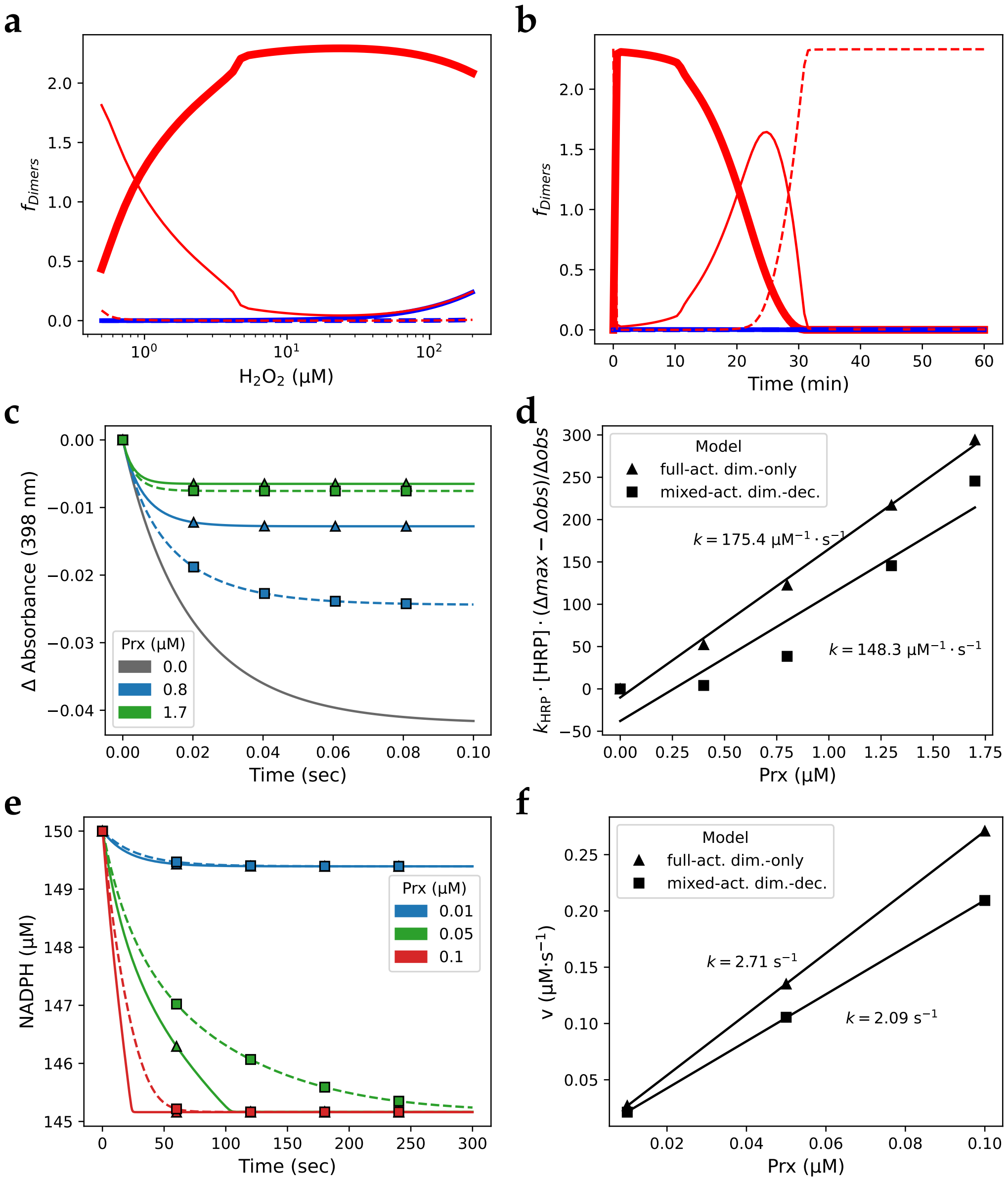

Figure S9: Including the formation of several disulphide bridges produces similar results to a model of rapid decamer disassembly after formation of a single disulphide-bridge. **(a)** and **(b)** Reproductions of the RBC simulations of **Figure 6 a&b**. **(c)** and **(d)** Reproductions of the HRP assay simulations of **Figure 7 a&b**. **(e)** and **(f)** Reproductions of the NADPH assay simulations of **Figure 7 c&d**.

References

1. Benfeitas, R., Selvaggio, G., Antunes, F., Coelho, P. & Salvador, A. Hydrogen peroxide metabolism and sensing in human erythrocytes: A validated kinetic model and reappraisal of the role of peroxiredoxin II. *Free Radical Biology and Medicine* **74,** 35–49 (2014).
2. Manta, B. *et al.* The peroxidase and peroxynitrite reductase activity of human erythrocyte peroxiredoxin 2. *Archives of Biochemistry and Biophysics* **484,** 146–154 (2009).
3. Peskin, A. *et al.* Hyperoxidation of peroxiredoxins 2 and 3: Rate constants for the reactions of the sulfenic acid of the peroxidatic cysteine. *Journal of Biological Chemistry* **288,** 14170–14177 (2013).
4. Urig, S., Lieske, J., Fritz-Wolf, K., Irmler, A. & Becker, K. Truncated mutants of human thioredoxin reductase 1 do not exhibit glutathione reductase activity. *FEBS Letters* **580,** 3595–3600 (2006).
5. Turanov, A., Su, D. & Gladyshev, V. Characterization of alternative cytosolic forms and cellular targets of mouse mitochondrial thioredoxin reductase. *Journal of Biological Chemistry* **281,** 22953–22963 (2006).
6. Barranco-Medina, S., Kakorin, S., Lázaro, J. & Dietz, K.-J. Thermodynamics of the dimer-decamer transition of reduced human and plant 2-cys peroxiredoxin. *Biochemistry* **47,** 7196–7204 (2008).
7. Tian, A. Recherches sur la calorimétrie. Généralisation de la méthode de compensation électrique. Microcalorimétrie. *Journal de Chimie Physique* **30,** 665–712 (1933).
8. Transtrum, M., Hansen, L. & Quinn, C. Enzyme kinetics determined by single-injection isothermal titration calorimetry. *Methods* **76,** 194–200 (2015).
9. Di Trani, J. M. *Measuring Enzyme Kinetics Using Isothermal Titration Calorimetry* PhD thesis (McGill University, Montreal, Canada, 2018).
10. Olivier, B. G., Rohwer, J. M. & Hofmeyr, J.-H. S. Modelling cellular systems with PySCeS. *Bioinformatics* **21,** 560–561 (Sept. 2005).
